# Application of quantile mixed-effects model in modeling CD4 count from HIV-infected patients in KwaZulu-Natal South Africa

**DOI:** 10.1186/s12879-021-06942-7

**Published:** 2022-01-04

**Authors:** Ashenafi A. Yirga, Sileshi F. Melesse, Henry G. Mwambi, Dawit G. Ayele

**Affiliations:** 1 School of Mathematics, Statistics, and Computer Science, University of KwaZulu-Natal, Pietermaritzburg, Private Bag X01, Scottsville, 3209 South Africa; 2 Institute of Human Virology, School of Medicine, University of Maryland, Baltimore, MD 21201 USA

**Keywords:** Quantile regression, Quantile mixed model, Stochastic approximation of the expectation maximization, Asymmetric Laplace distribution, CD4 count, CAPRISA

## Abstract

**Background:**

The CD4 cell count signifies the health of an individual’s immune system. The use of data-driven models enables clinicians to accurately interpret potential information, examine the progression of CD4 count, and deal with patient heterogeneity due to patient-specific effects. Quantile-based regression models can be used to illustrate the entire conditional distribution of an outcome and identify various covariates effects at the respective location.

**Methods:**

This study uses the quantile mixed-effects model that assumes an asymmetric Laplace distribution for the error term. The model also incorporated multiple random effects to consider the correlation among observations. The exact maximum likelihood estimation was implemented using the Stochastic Approximation of the Expectation–Maximization algorithm to estimate the parameters. This study used the Centre of the AIDS Programme of Research in South Africa (CAPRISA) 002 Acute Infection Study data. In this study, the response variable is the longitudinal CD4 count from HIV-infected patients who were initiated on Highly Active Antiretroviral Therapy (HAART), and the explanatory variables are relevant baseline characteristics of the patients.

**Results:**

The analysis obtained robust parameters estimates at various locations of the conditional distribution. For instance, our result showed that baseline BMI (at $\tau =$ 0.05: ${\widehat{\beta }}_{4}=0.056, \mathrm{p-}\mathrm{value}<0.0064; \mathrm{at }\,\tau = 0.5: {\widehat{\beta }}_{4}=0.082, \mathrm{p-}\mathrm{value}<0.0025; \mathrm{at}\,\tau = 0.95: {\widehat{\beta }}_{4}=0.145,\mathrm{p-}\mathrm{value}<0.0000$), baseline viral load (at $\tau =$ 0.05: ${\widehat{\beta }}_{5}$ $=-0.564, \mathrm{p-}\mathrm{value}<0.0000; \mathrm{at}\,\tau = 0. 5: {\widehat{\beta }}_{5}=-0.641, \mathrm{p-}\mathrm{value}<0.0000; \mathrm{at }\,\tau = 0.95: {\widehat{\beta }}_{5}=-0.739,\mathrm{p-}\mathrm{value}<0.0000$), and post-HAART initiation (at $\tau =$ 0.05: ${\widehat{\beta }}_{6}=1.683,\mathrm{p-}\mathrm{value}<0.0000; \mathrm{at}\,\tau = 0.5: {\widehat{\beta }}_{6}=2.560,\mathrm{p-}\mathrm{value}<0.0000; \mathrm{at }\,\tau =0.95: {\widehat{\beta }}_{6}=2.287,\mathrm{p-}\mathrm{value}<0.0000$) were major significant factors of CD4 count across fitted quantiles.

**Conclusions:**

CD4 cell recovery in response to post-HAART initiation across all fitted quantile levels was observed. Compared to HIV-infected patients with low viral load levels at baseline, HIV-infected patients enrolled in the treatment with a high viral load level at baseline showed a significant negative effect on CD4 cell counts at upper quantiles. HIV-infected patients registered with high BMI at baseline had improved CD4 cell count after treatment, but physicians should not ignore this group of patients clinically. It is also crucial for physicians to closely monitor patients with a low BMI before and after starting HAART.

**Supplementary Information:**

The online version contains supplementary material available at 10.1186/s12879-021-06942-7.

## Background

CD4 cell counts indicate a sign of the wellbeing of the immune system for an individual. It also provides information about disease progression. The number of CD4 cells of an individual who does not have HIV could be somewhere in the range of 500 to 1500 cells/mm^3^. “Individuals living with HIV who have a CD4 count above 500 cells/mm^3^ are usually in good health. Individuals living with HIV who have a CD4 cell count less than 200 cells/mm^3^ are at high risk of developing severe sickness” [1]. HIV therapy is recommended for all individuals infected with HIV. It is particularly critical for patients with low CD4 count to preferably starting treatment sooner rather than later, under the current WHO recommendation for individuals who test HIV positive [2].

The classical regression model about the mean has been the commonly applied statistical procedure to depict the effects of explanatory variables for continuous outcomes. Despite this, such results based on a fixed location of the response distribution may not be relevant in many areas, and sometimes the fields of application are diverse. Numerous investigators, economic experts, monetary stakeholders, clinicians, and legislators have revealed a growing interest in group differences across the whole population instead of relying solely on the average [3–6]. Another approach to studying the central location is median regression. The median regression approach is robust to the manifestation of outliers and when the error distribution is not correctly specified [3, 7].

Quantile regression (QR) was popularized by Koenker and Bassett [7]. It is an extension of median regression to examine the covariates’ influence on different quantiles of the entire response distribution. Fixed effects could have different impacts across various quantile levels. QR allows for a wide range of applications, for example, investigating the 5th or 25th percentile (lower quantiles) of the response (e.g., CD4 count distribution of HIV infected patient) might be of interest in studying patients with lower CD4 cell counts, where individuals are at higher risk of developing illnesses. Therefore, it is important to study the response distribution across all quantiles (e.g., at different CD4 count distribution), rather than only the central tendency, such as in mean or median regression.

In recent years, mixed quantile regression models have become a widely used technique in statistical studies. By using quantile-based regression model, it is possible to examine the location, scale, and shape of the distribution of responses to get an idea of how the covariates affect the distribution of responses. It is also more robust to outliers when compared to conventional mean regression and is invariant to monotonic transformations. There is no need to make any Gaussian assumptions concerning the response with quantile regression, and further it is capable of handling heavy-tailed and asymmetric data. As a result, CD4 count can be modeled very well using this method.

Many longitudinal studies gather a great deal of information about repeated measures that are crucial for analyzing disease progression in clinical studies. For example, repeated counts of CD4 cells are vital to HIV/AIDS monitoring; for instance, low levels of CD4 counts are signs of serious viral load accumulation, disease progression, and the need for therapy intervention. Physicians also use them to identify the advantages of medical involvement and the risk factors that may lead to poor outcomes. In practical statistics, mixed-effects models have become quite popular. As a result of their ability to handle both between-subjects and within-subjects variability in longitudinal data, they are often used to analyze longitudinal data [8]. Mixed-effects models and their estimated effects are formulated on the response variable via mean regression, accounting for between-subject heterogeneity through normally distributed subject-specific random effects and random errors. Mixed-effects models have been studied extensively (see, for example, [8–12]). There are also various strategies applicable to handle longitudinal data, for instance, generalized estimating equations which are conceptually generalized linear mixed-effects models. However, all these techniques limit the investigation of variations between subjects based on the mean of the response variable, and the latter utilize parametric models based on the normal distributional assumption [3].

Moreover, in some cases, it could be challenging to obtain appropriate transformation to normality for the response variable, or some strategy to account for outliers may be required. An adequate solution to all these issues is given by concentrating on the conditional quantiles of the longitudinal outcome [13]. “Conditional QR methods, dealing with the complete conditional distribution of the response variable, have been developed to grant an analysis of variable effects at any subjective quantiles of the response distribution. Furthermore, QR techniques do not require any distributional assumption on the error; besides that, the error term has a zero-conditional quantile, like the ALD” [14].

The QR method was initially developed in a univariate setting. However, the large amount of longitudinal data has recently dictated its extension into a mixed-effects modeling system by either the distribution-free way [15–17] or the likelihood-based way in most cases following the ALD [18–21]. The likelihood-based quantile mixed model additionally makes use of different parametric distributions, such as an infinite mixture of Gaussian densities [22] and a direct parametric maximum likelihood (ML) approach [23]. The distribution-free approaches that consist of fixed-effects and weighted generalized estimating equations consider the use of *independent* estimating equations that ignore correlations between repeated measurements which leads to loss of efficiency [5, 17, 20]. Meanwhile, Geraci and Bottai [19] suggested a likelihood-based QR model for longitudinal data that accounts for within-subject dependence by incorporating subject-level random effects and modeling the residual distribution with an ALD. Liu and Bottai [24] developed a likelihood-based method to estimate parameters of conditional quantile functions with random effects by incorporating an ALD for the random error term that is not restricted to be normal. The within-subject correlation is taken into consideration by incorporating random effects to get unbiased parameter estimates [24]. The application of QR for mixed-effects models has received increasing consideration in wide-ranging areas of study, including marine biology, environmental science, cardiovascular disease, and ophthalmology [19, 20, 25–28]. Following the version of the quantile mixed model of Galarza [18], this study aims to model the longitudinal CD4 count of HIV-infected patients using quantile mixed-effects models using the likelihood-based function that uses ALD for the error term. The study employs data from the CAPRISA 002 AI Study. In this study, we will demonstrate how quantile mixed model can be used to estimate covariate effects at different locations of the conditional distribution that communicates a wide-range and more complete picture of the effects.

## Methods

### Data description

This study used the Centre of the AIDS Programme of Research in South Africa (CAPRISA) 002 Acute Infection (AI) Study data conducted at the Doris Duke Medical Research Institute (DDMRI) at the Nelson R Mandela School of Medicine of the University of KwaZulu-Natal (UKZN) in Durban, South Africa [29–33]. CAPRISA started the CAPRISA 002 AI study between August 2004 and May 2005 by enrolling women who are at high risk of HIV infection for follow-up with an intense on-going examination to help estimate HIV infection rates within the study, including providing intense aftercare advice to those dropping out prematurely, the careful follow-up to study disease progression, and CD4 count and viral load evolution [29–33]. Detail description of the design, development, and procedures of the CAPRISA 002 AI study population can be found here [29, 30].

When an infected person’s body indicates symptoms of being incapable of adequately controlling the virus and their CD4 count drops below a specific cut point, they were initiated on therapy. A deficient level of CD4 count causes the weak immune system of an HIV-infected person. In the absence of treatment or without viral suppression, the person is susceptible to opportunistic infections (OIs). This increases the risk of the new and ongoing Coronavirus Disease 2019 (COVID-19) infections and underlying illnesses [31–33]. HAART is an effective way of preventing these infections and diseases. By suppressing and preventing the virus from making copies of itself, HAART aims to decelerate or prevent the progression to AIDS and loss of life for HIV-infected people. The body’s immune system is less damaged, and HIV infection complications are decreased when the level of the virus in the blood is low or “undetectable” through HAART [31–33]. This is also significantly reducing the likelihood of transmitting HIV to partners.

The HIV/AIDS epidemic and other sexually transmitted diseases severely impact human health, especially the well-being of women and young girls [31–33]. “The consequences of HIV/AIDS stretch beyond women’s health to their part as moms and caregivers and their commitment to their families’ economic support. The social, development, and health consequences of HIV/AIDS and other sexually transmitted illnesses ought to be considered from a gender perspective” [34–36]. Apart from sex-specific issues, HIV therapy algorithms for women are similar to that of men [31]. The interaction between the clinician and the changing HIV epidemiology will provide the clinician with a technique to identify patients at high risk of HIV infection and clarify which rules should be applied to avoid sequential HIV transmission [31–33]. Although ART suggestions are the same for all patients, the study of CD4 count of HIV-infected patients, in conjunction with individual differences, will help clinicians to get through and interpret potential information precisely due to patient specific-specific effects [31, 33, 37–39].

### Quantile mixed-effects model

Quantile regression (QR) is an advanced statistical technique to study the predictors’ heterogeneous effects at the conditional distribution of the outcome. Instead of modeling only the mean value like the conventional regression methods, quantile regression enables more fully to explore the data by modeling the conditional quantiles, for example, the 5th and 95th percentiles of the response distribution [33]. For these reasons, it has become more prevalent in several epidemiological and economics studies. For instance, Yirga et al. [40] studied how children’s BMI varies with age and other factors using quantile regression. There are several other applications of quantile regression based on uncorrelated data, among which public health, bioinformatics, health care, environmental science, ecology, microarray data analysis, and survival data analysis [13, 41–51].

The quantile level is frequently signified by the Greek letter $\tau$, and the conditional quantile of $y$ given $x$ is often written as ${Q}_{\tau }(y|x)$. The quantile level $\tau$ is the probability $\mathrm{Pr}[y\le {Q}_{\tau }(y|x)]$, and it is the value of $y$ below which the proportion of the conditional response population is $\tau$. For a random variable $y$ with a probability distribution function $F\left(y\right)=Pr\left(Y\le y\right)$, the τ quantile of $y$ is defined as the inverse function $Q\left(\uptau \right)=inf\left\{y:F(y)\ge\uptau \right\}$, $\uptau \upepsilon (0, 1)$. Particularly, the median is $Q\left(0.5\right)$. Let ${y}_{i}$ denote a scalar response variable with conditional cumulative distribution function ${F}_{{y}_{i}}$, whose shape is unspecified and ${{\varvec{x}}}_{i}$ the corresponding covariates vector of dimension $k\times 1$ for subject $i, i=1,\dots ,n$. Then, following Koenker and Basset (1978), the $\tau \mathrm{th}(0<\tau <1)$ quantile regression modeled is written as ${Q}_{\tau }\left({y}_{i}|{{\varvec{x}}}_{{\varvec{i}}}\right)={{\varvec{x}}}_{{\varvec{i}}}^{\boldsymbol{^{\prime}}}{{\varvec{\beta}}}_{{\varvec{\tau}}}$, where ${Q}_{\tau }\left({y}_{i}|{{\varvec{x}}}_{{\varvec{i}}}\right)\equiv {F}_{{y}_{i}}^{-1}\left(\bullet \right)$, which is the quantile function (or the inverse cumulative distribution function) of ${y}_{i}$ given ${{\varvec{x}}}_{{\varvec{i}}}$ estimated at $\tau$, and ${{\varvec{\beta}}}_{\tau }$ is a column vector of regression parameters corresponding to the $\tau \mathrm{th}$ quantile. On the other hand, this expression can be written as

1 $${Q}_{\tau }\left(y|{{\varvec{x}}}_{{\varvec{i}}}\right)={{\varvec{x}}}_{{\varvec{i}}}^{\mathbf{^{\prime}}}{{\varvec{\beta}}}_{{\varvec{\tau}}}+{\varepsilon }_{i},\mathrm{ with }\,{Q}_{{\varepsilon }_{i}}\left(\tau |{{\varvec{x}}}_{i}\right)=0,$$

where ${\varepsilon }_{i}$ is the error term whose distribution (with density ${f}_{\tau }\left(\bullet \right)$) is restricted to have the $\tau \mathrm{th}$ quantile to be zero, that is, ${\int }_{-\infty }^{0}{f}_{\tau }\left({\varepsilon }_{i}\right)d{\varepsilon }_{i}=\tau$ [24, 52]. “The error density ${f}_{\tau }\left(\bullet \right)$ is often left unspecified in the classical literature” [52]. Thus, the estimator ${\widehat{{\varvec{\beta}}}}_{\tau }$ proceeds through *linear programming (LP)* by minimizing

2 $${\widehat{{\varvec{\beta}}}}_{\tau }=\underset{\mathit{\beta \epsilon }{R}^{P}}{\mathrm{argmin}}{\sum }_{i=1}^{n}{\rho }_{\tau }({y}_{i}-{{\varvec{x}}}_{{\varvec{i}}}^{\mathbf{^{\prime}}}{{\varvec{\beta}}}_{{\varvec{\tau}}}),$$

where ${\rho }_{\tau }\left(\bullet \right)$ is the so called loss (or check) function defined by ${\rho }_{\tau }\left(u\right)=u\left(\tau -I\left\{u<0\right\}\right)$ with $u$ being a real number and $I\left\{\bullet \right\}$ is the indicator function. Thus, ${\widehat{{\varvec{\beta}}}}_{\tau }$ is called the $\tau \mathrm{th}$ quantile regression estimate [5, 13, 43, 53]. The parameter ${{\varvec{\beta}}}_{\tau }$ and its estimator ${\widehat{{\varvec{\beta}}}}_{\tau }$ depends on the quantile $\tau$, because of different choices of $\tau$ estimate different values of $\beta$ [24]. For this reason, the interpretation of ${{\varvec{\beta}}}_{\tau }$ is specific to the quantile being estimated, the intercept term denotes the baseline predicted value of the response at specific quantile $\tau$, while each coefficient can be interpreted as the rate of change of the $\tau \mathrm{th}$ response quantile per unit change in the value of the corresponding predictor variable (*i*th regressor) keeping all the other covariates constant.

The objective function of the conditional quantile estimator, ${\widehat{{\varvec{\beta}}}}_{{\varvec{\tau}}}$, in Eq. (2) proceeds by minimizing

3 $$\begin{aligned}H\left({{\varvec{\beta}}}_{{\varvec{\tau}}}\right) & =\sum_{i}\tau \left|{\varepsilon }_{i}\right|+\sum_{i}\left(1-\tau \right)\left|{\varepsilon }_{i}\right|\\&=\sum_{i:{y}_{i}\ge {{\varvec{x}}}_{{\varvec{i}}}^{\boldsymbol{^{\prime}}}{{\varvec{\beta}}}_{{\varvec{\tau}}}}^{n}\tau |{y}_{i}-{{\varvec{x}}}_{{\varvec{i}}}^{\boldsymbol{^{\prime}}}{{\varvec{\beta}}}_{{\varvec{\tau}}}|+\sum_{i:{y}_{i}<{{\varvec{x}}}_{{\varvec{i}}}^{\boldsymbol{^{\prime}}}{{\varvec{\beta}}}_{{\varvec{\tau}}}}^{n}(1-\tau )|{y}_{i}-{{\varvec{x}}}_{{\varvec{i}}}^{\boldsymbol{^{\prime}}}{{\varvec{\beta}}}_{{\varvec{\tau}}}|,0< \tau <1 \end{aligned}$$

where $i:{y}_{i}\ge {{\varvec{x}}}_{{\varvec{i}}}^{\boldsymbol{^{\prime}}}{\varvec{\beta}}$ for under prediction, and $i:{y}_{i}<{{\varvec{x}}}_{{\varvec{i}}}^{\boldsymbol{^{\prime}}}{\varvec{\beta}}$ for overprediction [5]. Since the above objective function is nondifferentiable, the gradient optimization methods are not applicable; instead, *LP* methods can be used to obtain $H({{\varvec{\beta}}}_{{\varvec{\tau}}})$ [41, 54, 55]. For more details and a summary of quantile regression, see, for example, Davino et al. [3], Konker and Basset [7], Konker [13], Buchinsky [41], Koenker and Hallock [43], or Yu et al. [49].

As the check function $({\rho }_{\tau }\left(\bullet \right))$ in Eq. (2) is not differentiable at zero, we cannot extract specific solutions to the minimization problem. Hence, *LP* procedures are often used to achieve a relatively fast computation of $H({{\varvec{\beta}}}_{{\varvec{\tau}}})$ [52, 56]. A natural link between minimization of the quantile check function and ML theory is given by the assumption that the error term in Eq. (1) follows an ALD [53, 57]. A connection between the minimization of the sum in Eq. (2) and the ML theory is provided by ALD [58]. Other forms of Laplace distribution were summarized by Kotz et al. [59] and Kozubowski and Nadarajah [60]. ALD that is closely associated with the loss function for QR has been examined in several works of literature [19, 24, 52, 57, 58].

The conventional QR is based on the median, or other quantile levels, by assuming a continuous or Gaussian distribution. QR has been extended to count regression, which is a special case of the discrete variable model [55, 56, 61–64]. However, the distribution function of a discrete random variable is not continuous, and the objective function of the conditional quantile ${Q}_{\tau }(y|{\varvec{x}})$ for a discrete distribution cannot be a continuous function of ${\varvec{x}}$ such as $\mathrm{exp}({{\varvec{x}}}^{\boldsymbol{^{\prime}}}{\varvec{\beta}})$ [61]. Machado and Silva [64] overcome this restriction by developing a continuous random variable whose quantiles have a one-to-one relation with the quantiles of $y$, a count variable. When count data consists of severe outliers or multiple distributional components that do not reflect a known underlying probability distribution, quantile count models may be a useful alternative. Furthermore, QR models all of the quantiles of the discrete distribution and covers the entire range of counts [62]. Detailed discussions about quantile count models for independent data are available in Winkelmann [61], Machado and Silva [64], Hilbe [62, 63], Cameron and Trivedi [55, 56], and a recent application of this model can be found in Winkelmann [65] and Miranda [66].

Mixed-effects models characterize an ordinary and conventional type of regression methods used to examine data coming from longitudinal studies. The general linear mixed-effects model is defined as

$${{\varvec{Y}}}_{i}={{\varvec{X}}}_{i}^{^{\prime}}{\varvec{\beta}}+{{\varvec{Z}}}_{i}^{^{\prime}}{{\varvec{u}}}_{i}+{\varepsilon }_{ij},\quad i=1,\dots ,n,\quad j=1,\dots ,{n}_{i},$$

where ${{\varvec{Y}}}_{i}$ is the ${n}_{i}\times 1$ vector of the response variable, ${{\varvec{X}}}_{i}^{^{\prime}}$ is a known ${n}_{i}\times p$ design matrix that includes covariates for the fixed effects, ${\varvec{\beta}}$ is $p\times 1$ vector of population-averaged fixed-effects, ${{\varvec{Z}}}_{i}^{^{\prime}}$ with the dimension of ${n}_{i}\times r$ known design matrix for random effects, ${{\varvec{u}}}_{i}$ is $r\times 1$ vector of random effects, ${{\varvec{u}}}_{i}\sim N\left(0, {\boldsymbol{\Sigma }}_{u}\right),$ and ${\varepsilon }_{ij}$ is the independent and identically distributed random errors, ${\varepsilon }_{ij}\sim N(0,{\sigma }^{2})$. Thus, the $\tau \mathrm{th}$ quantile linear mixed-effects model, which were developed by Geraci and Bottai [20] as an extension of the QR model with a random intercept of Geraci and Bottai [19], of a continuous response ${{\varvec{Y}}}_{i}$, has the form

4 $${Q}_{\tau }\left({y}_{ij}|{{\varvec{x}}}_{ij},{\boldsymbol{ }{\varvec{u}}}_{i}\right)={{\varvec{x}}}_{ij}^{^{\prime}}{{\varvec{\beta}}}_{\tau }+{{\varvec{z}}}_{ij}^{^{\prime}}{{\varvec{u}}}_{i}+{\varepsilon }_{\tau ,ij}, 0<\tau <1$$

where ${y}_{ij}$ is the response of subject $i$ at $j$th measurement, ${{\varvec{x}}}_{ij}$ indicates covariate vector of $i$th subject at $j$th measurement for fixed effects, ${{\varvec{z}}}_{ij}$ indicates covariate vector of $i$th subject at $j$th measurement for the random effects ${{\varvec{u}}}_{i}$, and random errors ${\varepsilon }_{\tau ,ij}\sim ALD(0,\sigma ,\tau )$, which are also dependent on $\tau$. ${{\varvec{\beta}}}_{\tau }$ is the coefficient of fixed-effects corresponding to the $\tau \mathrm{th}$ quantile, and the response variable ${y}_{ij}$, conditional on ${{\varvec{x}}}_{ij}$, ${{\varvec{u}}}_{i}$, for $i=1,\dots ,n, j=1,\dots ,{n}_{i}$ and $\sigma$ are assumed to be conditionally independently distributed as ALD with the density given by

5 $$f\left({y}_{ij}|{{\varvec{x}}}_{ij},{{\varvec{u}}}_{i}, \sigma \right)=\frac{\tau \left(1-\tau \right)}{\sigma }exp\left\{-{\rho }_{\tau }\left(\frac{{y}_{ij}-{{\varvec{x}}}_{ij}^{\mathrm{^{\prime}}}{{\varvec{\beta}}}_{\tau }-{{\varvec{z}}}_{ij}^{\mathrm{^{\prime}}}{{\varvec{u}}}_{i}}{\sigma }\right)\right\}.$$

The random effects (${{\varvec{u}}}_{i}$’s) are assumed to be distributed as ${{\varvec{u}}}_{i}\stackrel{iid}{\sim }{N}_{r}\left(0,\boldsymbol{\Psi }\right)$, where the dispersion matrix $\boldsymbol{\Psi }=\boldsymbol{\Psi }(\boldsymbol{\alpha })$ relies on unknown and reduced parameters $\boldsymbol{\alpha }$, which is the distinct elements of $\boldsymbol{\Psi }$, and the random errors ${\varepsilon }_{ij}\sim ALD(0,\sigma )$ [18, 52]. Then a likelihood for ${y}_{ij}$ at $\tau \mathrm{th}$ quantile is

6 $$L\left({{\varvec{\beta}}}_{\tau },\sigma ,\tau \right)=\frac{{\tau }^{n}{\left(1-\tau \right)}^{n}}{{\sigma }^{n}}exp\left\{-\sum_{i=1}^{n}{\sum }_{j=1}^{{n}_{i}}{\rho }_{\tau }\left(\frac{{y}_{ij}-{{\varvec{x}}}_{ij}^{^{\prime}}{{\varvec{\beta}}}_{\tau }-{{\varvec{z}}}_{ij}^{^{\prime}}{{\varvec{u}}}_{i}}{\sigma }\right)\right\}$$

Based on the likelihood of conditional quantile of ${y}_{ij}$, it is suggested that the maximization of the likelihood in Eq. (5) with respect to the parameter ${{\varvec{\beta}}}_{\tau }$ is equivalent to the minimization of the loss function in Eq. (7). Thus, we can estimate the coefficient of fixed-effects corresponding to the $\tau \mathrm{th}$ quantile (${{\varvec{\beta}}}_{\tau }$) by minimizing the objective function of Eq. (6), which can be expressed as

7 $${H}^{*}({{\varvec{\beta}}}_{{\varvec{\tau}}})=\underset{{{\varvec{\beta}}}_{\tau }}{\mathrm{min}}{\sum }_{i=1}^{n}{\sum }_{j=1}^{{n}_{i}}{\rho }_{\tau }\left(\frac{{y}_{ij}-{{\varvec{x}}}_{ij}^{^{\prime}}{{\varvec{\beta}}}_{\tau }-{{\varvec{z}}}_{ij}^{^{\prime}}{{\varvec{u}}}_{i}}{\sigma }\right)$$

More details regarding the estimation process of quantile mixed-effects models are available here [18, 19, 24, 58].

### Stochastic approximation of the expectation maximization

The study examines quantile regression for linear mixed-effects models (QR-LMM) of Galarza [18] that follows the SAEM algorithm for determining exact ML estimates of the fixed-effects and the general variance–covariance matrix ${\boldsymbol{\Sigma }}_{\tau }=\boldsymbol{\Sigma }\left({{\varvec{\theta}}}_{{\varvec{\tau}}}\right)$ of the random effects parameters for the specific quantile. The Expectation–Maximization algorithm, also known as the EM algorithm, which was suggested by Dempster et al. [67], is a popular technique for iterative computation of ML estimates when the observations are regarded as incomplete data, which incorporates the ordinary or standard elements of missing data; however, it is much broader than that [68]. There are two steps in every iteration of the EM algorithm: an expectation, or E-step, followed by a maximization (M-step). “In the former action, the incomplete data are estimated given the observed data and current estimate of the model parameters under the assumption of missing at random (MAR) for the incomplete data. In the later step, the likelihood function is maximized under the assumption that the incomplete/missing data is known” [67]. The detailed explanations of these processes, their related analytical clarifications for successively more common sorts of models, and the basic theory underlying the EM algorithm are given by Dempster et al. [67]. A book devoted entirely to the general formulation of the EM algorithm and its basic properties and applications has been provided by McLachlan and Krishnan [68]. Moreover, the success of the EM algorithm is well documented and can be found in numerous statistical literature.

Even though the EM algorithm is popular, Delyon et al. [69] pointed out that, in some situations, it is not applicable due to the fact that the E-step cannot be carried out in a closed-form. To deal with these issues, Delyon et al. [69] presented a simulation-based SAEM algorithm based on stochastic approximation (SA) as an elective to the MCEM, standing for Monte Carlo EM. “While the MCEM requires a consistent increment of the simulated data and regularly a substantial number of simulations, the SAEM versions guarantee convergence with a fixed and/or small simulation size” [69–71]. The SAEM algorithm restores the E-step of the EM algorithm by one iteration of a stochastic (probabilistic) approximation procedure, whereas the M-step is consistent [71]. The E- and M-steps of the EM and SAEM procedures are highlighted as follows.

Let ${\mathcal{l}}_{o}\widehat{(\uptheta })=\mathrm{log}f({Y}_{obs};\uptheta )$ denotes the maximization of log-likelihood function based on the observed data $({Y}_{obs})$, and given $q$ represents missing data, ${Y}_{com}=({Y}_{obs}, q){^{\prime}}$ denotes the complete data with observed and missing data, thus ${\mathcal{l}}_{c}({Y}_{com};\uptheta )$ be the complete log-likelihood function, and ${\widehat{\uptheta }}_{k}$ indicates the evaluation of $\uptheta$ at the $k$th iteration. Then the EM algorithm with missing data that maximizes ${\mathcal{l}}_{c}\left({Y}_{com};\uptheta \right)=\mathrm{log}f({Y}_{obs}, q;\uptheta )$ iteratively and converges to a stationary point of the observed likelihood under mild regularity conditions [18, 71], go through in two steps:

- E-step: Consists computing of the conditional expectation of ${\mathcal{l}}_{c}({Y}_{com};\uptheta )$.
  $$S\left(\uptheta |{\widehat{\uptheta }}_{k}\right)=E\left\{{\mathcal{l}}_{c}\left({Y}_{com};\uptheta \right)|{Y}_{obs}, {\widehat{\uptheta }}_{k}\right\}$$
- M-step: Computes the parameter values ${\widehat{\uptheta }}_{k+1}$ by maximizing $S\left(\uptheta |{\widehat{\uptheta }}_{k}\right)$ with respect to $\uptheta$.
  The SAEM algorithm, on the other hand replaces the E-step by stochastic approximation, presented by Galarza [18] summarized as follows:
- Simulation (E-step): Generate $q({\mathcal{l}}_{o}, k)$ sample (simulation of the missing data at iteration $k$), $\mathcal{l}=\mathrm{1,2},\dots , m$, from the conditional distribution of the missing data $f\left(q|{\uptheta }_{k-1}, {Y}_{obs}\right)$.
- Stochastic approximation: Update $S\left(\uptheta |{\widehat{\uptheta }}_{k}\right)$ according to
  $$S\left(\uptheta |{\widehat{\uptheta }}_{k}\right)=S\left(\uptheta |{\widehat{\uptheta }}_{k-1}\right)+{\delta }_{k}\left[\frac{1}{m}\sum_{\mathcal{l}=1}^{m}{\mathcal{l}}_{c}\left({Y}_{obs},q\left({\mathcal{l}}_{o}, k\right)|{\widehat{\uptheta }}_{k};\uptheta \right)-S\left(\uptheta |{\widehat{\uptheta }}_{k-1}\right)\right]$$
- M-step: Maximize ${\widehat{\uptheta }}_{k}$ according to
  $${\widehat{\uptheta }}_{k+1}=\underset{\uptheta }{\mathrm{argmax}}S\left(\uptheta |{\widehat{\uptheta }}_{k}\right),$$

this is equivalent to finding ${\widehat{\uptheta }}_{k+1} \upepsilon {\varvec{\Theta}}$ such that $S\left({\widehat{\uptheta }}_{k+1}\right)\ge S\left({\widehat{\uptheta }}_{k}\right)$∀$\uptheta \upepsilon {\varvec{\Theta}}$, where ${\delta }_{k}$ is a smoothing parameter (a sequence of decreasing non-negative numbers) as given by Kuhan and Lavielle [72, 73], and $m$ is the number of simulations suggested to be less than or equal to 20 [18]. The choice of ${\delta }_{k}$ recommended by Galarza [18] is given as follows:

$${\delta }_{k}=\left\{\begin{array}{l}1\, for\, 1\le k\le cW\\ \frac{1}{k-cW}\, for\, cW+1\le k\le W ,\end{array}\right.$$

where $c \upepsilon (0, 1)$ is a cut point that regulates the percentage of initial iterations with no memory, and $W$ is the maximum number of iterations.

For more points of interest, however, see Jank [70], Meza et al. [71], or Kuhn and Lavielle [72, 73]. Furthermore, details of these algorithms for estimating the parameters of the QR-LMM are presented by Galarza [18] and Galarza et al. [21]. “The SAEM algorithm has proven to be more effective for computing the ML estimates in mixed-effects models due to the reusing of simulations from one iteration to the next in the smoothing phase of the algorithm” [18, 71–73]. The SAEM algorithm is employed in the R package *qrLMM*.

## Results

CD4 cells are the utmost target of HIV infection, and the CD4 count is used as a health marker for an individual’s immune system. Hence, it is of interest to investigate the evolution of CD4 count and disease progression of an individual over time, especially for HIV-infected patients. Consequently, this study analyzes the repeated CD4 count of HIV-positive patients registered in the CAPRISA 002 AI study by employing a parametric quantile regression mixed-effects model based on the asymmetric Laplace distribution. The CAPRISA 002 AI study dataset consists of repeated CD4 count measurements and some other covariates of 235 individuals. There were a total of 7019 observations from the 235 women; each subject was measured several times, ranging from 2 to 61 months, with a median equal to 29. Table 1 illustrates a summary of the patients’ baseline characteristics. The patients’ age at enrollment ranges from 18 to 59, with the median age being 25 years. ${Q}_{0.05}$, which is a value that has 5% of the observation smaller or equal to it, indicates that 5% of the patients had a square root of CD4 count below or equal to 16.4 at enrollment. ${Q}_{0.95}$ is similarly a value that shows 95% of the observation smaller or equal to it; said otherwise, 5% of the patients are greater than it. Therefore, Table 1 indicates 5% of the study participant had a square root CD4 count greater than 31.4 at enrollment. Moreover, the study participants had a mean BMI of 28.93 with minimum and maximum BMI of 17.89 and 54.89 at baseline. The median log baseline VL of the patients was 10.26 with minimum and maximum log baseline VL of 0 (Not detected) and 15.52, respectively (IQR = 2.91). Additional features on this dataset can be found here [29, 30, 32, 33]. We analyze this dataset intending to explain the different conditional distribution of the square-root-transformed CD4 count as a function of sets of covariates of interest through modeling a framework of response quantiles.

**Table 1:** Summary of patients’ baseline characteristics

Variable	Analysis						
	Mean	Median	Minimum	Maximum	Q_0.05_	Q_0.95_	IQR
SQRT_CD4 count	23.44	22.89	13.49	39.49	16.40	31.40	5.78
Baseline BMI	28.93	27.24	17.89	54.89	20	43.70	9.66
Log_Baseline VL	10.09	10.26	0 (undetected)	15.52	6.19	13.13	2.91
Age at baseline	27.15	25	18	59	20	41	8

The linear mixed-effects model form of the data can be specified as:

$${y}_{ij}={\beta }_{1}+{\beta }_{2}{t}_{i}+{\beta }_{3}\sqrt{{t}_{i}}+{\beta }_{4}{BMI}_{i}+{\beta }_{5}{LVL}_{i}+{\beta }_{6}{ART}_{i}+{\beta }_{7}{Age}_{i}+{b}_{1i}+{b}_{2i}{t}_{i}+{b}_{3i}\sqrt{{t}_{i}}+{\varepsilon }_{ij,}$$

where ${y}_{ij}$ is the transformed continuous form of CD4 count ($\sqrt{CD4} \,count$) at the *j*th time point for the *i*th subject, $t$ is the time measured in months from the start of the study, BMI indicates the patient’s baseline BMI, LVL = log of baseline VL, ART is the dichotomous HAART initiation (0 = pre-HAART, 1 = post-HAART), Age is patient’s age at baseline, ${b}_{1i}$ indicates the random intercept, ${b}_{2i}$ and ${b}_{3i}$ indicates the random slopes (for time and square root time respectively) for subject $i$, and ${\varepsilon }_{ij}$ the measurement error term, assuming ALD, for 235 subjects.

The information criteria are used to compare four models. The models were compared based on the 0.5th quantile (median regression). The linear quantile mixed-effects model with random intercept and slopes (Model 4, see Table 2) was selected as the best model because the chosen model achieved the smallest Akaike information criteria (AIC), Bayesian information criteria (BIC), Hannan–Quinn information criteria (HQC), and the largest Log-likelihood (LL) (see Table 2). Therefore, we examine the square-root-transformed CD4 count of HIV-infected patients as a response while accounting for Baseline BMI, age, log baseline VL, and HAART initiation as predictor variables across various quantiles based on Model 4 (Table 3). A series of QR-LMM at $\tau =0.05, 0.25, 0.5, 0.75, 0.85$, and $0.95$ are performed to get a complete picture of the effects (see, Table 3, and Additional files 1, 2).

**Table 2:** Comparison of random effects models for QR-LMM at the 0.5th quantile

Random effects	AIC	BIC	HQC	LL
Model 1	39,670.99	39,725.84	39,689.89	− 19,827.50
Model 2	35,072.84	35,141.41	35,096.47	− 17,526.42
Model 3	35,726.22	35,794.79	35,749.85	− 17,853.11
Model 4	33,685.92	33,781.91	33,718.99	− 16,828.96

**Table 3:** Parameter estimates for CAPRISA 002 AI study data across several quantiles

Parameter	${\widehat{Q}}_{0.05}$ (SE)	${\widehat{Q}}_{0.25}$ (SE)	${\widehat{Q}}_{0.5}$ (SE)	${\widehat{Q}}_{0.75}$ (SE)	${\widehat{Q}}_{0.85}$ (SE)	${\widehat{Q}}_{0.95}$ (SE)
Intercept	19.996 (1.161)*	22.171 (1.403)*	24.628 (1.464)*	26.595(1.419)*	27.972 (1.420)*	31.381 (1.397)*
Time	0.063 (0.015)*	0.069 (0.013)*	0.056 (0.013)*	0.046 (0.013)*	0.041 (0.013)*	0.034 (0.015)*
SQRT of time	− 0.866 (0.142)*	− 0.871 (0.129)*	− 0.695 (0.117)*	− 0.593 (0.119)*	− 0.581 (0.124)*	− 0.385 (0.162)*
Baseline BMI	0.056 (0.021)*	0.078 (0.024)*	0.082 (0.026)*	0.112 (0.032)*	0.131 (0.033)*	0.145 (0.030)*
Log of baseline VL	− 0.564 (0.078)*	− 0.568 (0.103)*	− 0.641 (0.096)*	− 0.713 (0.093)*	− 0.714 (0.089)*	− 0.739 (0.084)*
Post HAART initiation	1.683 (0.054)*	2.125 (0.073)*	2.560 (0.088)*	3.021 (0.096)*	3.114(0.097)*	2.287 (0.089)*
Age	0.021 (0.025)	0.029 (0.029)	0.029 (0.031)	0.029 (0.032)	0.026 (0.032)	0.013 (0.030)
Log-lik	− 18,454.68	− 17,169.85	− 16,828.96	− 17,344.63	− 17,952.50	− 19,088.77
AIC	36,937.36	34,367.69	33,685.92	34,717.25	35,933	38,205.55

^*^Significance at 5% level. See, Additional file 1, for more significant test results and confidence intervals

*Random effect models that were examined for the analysis*

$$\begin{array}{*{20}l} {\text{Model 1: Time}} & {\text{(Random slope model )}} \\ {\text{Model 2: Intercept, Time}} & {\text{(Random intercept and slope model )}} \\ {{\text{Model 3: Time, }}\sqrt {Time} } & {\text{(Random slopes model )}} \\ {{\text{Model 4: Intercept, Time, }}\sqrt {Time} } & {\text{(Random intercept and slopes model )}} \\ \end{array}$$

As can be observed from Table 3, the intercept (${\beta }_{1}$), which is the predicted value of the square-root-transformed CD4 count keeping all the other covariates zero, differ significantly across the quantiles, while time (${\beta }_{2}$), square root of time (${\beta }_{3}$), baseline BMI $({\beta }_{4})$, the log of baseline VL (${\beta }_{5}$), and post HAART initiation (${\beta }_{6}$) significantly affect the CD4 count across all quantiles. In addition, although age (${\beta }_{7}$) is found to have a positive and almost constant influence on the CD4 count across all quantiles, its effect is non-significant (Table 3). We can also see from Table 3; there is a remarkable positive effect of baseline BMI on square root CD4 cell count ($\sqrt{CD4} \,count$) from low quantiles to higher quantiles. Whereas, from low to more upper quantiles, the negative effect of baseline VL on the count of CD4 cells increases gradually. This indicates that when the VL at enrollment is high (baseline VL at higher quantiles), its negative effect on the immune systems increases (Table 3). From low quantiles to upper quantiles, the post HAART initiation effect on CD4 cell counts has an increasing trend, and then at high quantile 0.95, its effect begins to decline.

R package *qrLMM()* sample outputs using CAPRISA 002 Acute Infection Study data across all fitted quantile levels can be found in Additional files 1, 2.

**Fig. 1:**
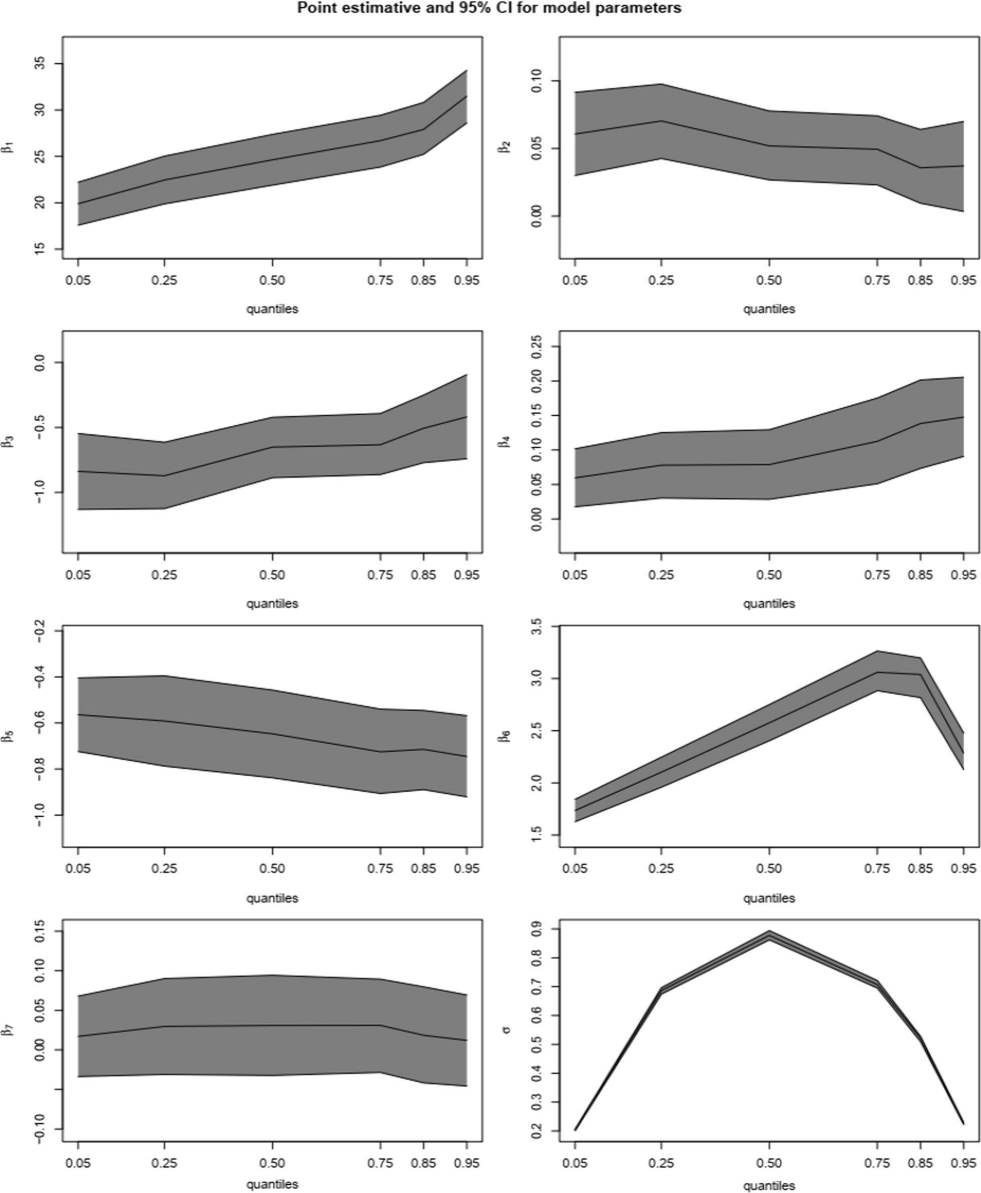
Point estimates and 95% confidence bands for model parameters following the QR-LMM to the CAPRISA 002 AI Study data across various quantiles

The results in graphical representation following QR-LMM over the framework of quantiles $\tau =\{0.05, 0.25, 0.5, 0.75, 0.85, 0.95\}$ are displayed in Fig. 1. The graph shows that the 95% confidence interval for the covariates effect and the nuisance parameter $\sigma$. The figure reveals that the effect of baseline BMI (${\beta }_{4}$), and post HAART initiation (${\beta }_{6}$) become more prominent across quantile levels, with their effect becoming more for higher conditional quantiles. Additionally, although the effects of time ${(\beta }_{2})$ and baseline VL (${\beta }_{5}$) exhibit a significant positive and negative influence, respectively, on CD4 counts across all quantiles, the difference changes with regard to the lower quantiles. The $\widehat{\sigma }$ is symmetric about $\tau =0.5$, taking its maximum value at that point and decreasing for higher quantiles. The convergence of estimates for all parameters was also evaluated using the graphical criteria (see Additional files 1, 2).

## Conclusions

This study considered a quantile mixed-effects model with a likelihood-based function that adopts an ALD for the error term. We used the SAEM algorithm for determining exact ML estimates of the covariates effect and variance–covariance elements across a set of quantiles. We applied this methodology to the CAPRISA 002 AI Study data and illustrated how the procedure can be used to obtain robust parameters estimates when the interest is to get the estimation not only on the central location but also on the non-central locations of the conditional distribution, which brings a comprehensive and more complete picture of the effects. A series of QR-LMM at $\tau =0.05, 0.25, 0.5, 0.75, 0.85$ and $0.95$ were estimated (Table 3, and Additional files 1, 2), and the results were discussed.

Since quantile inference for discrete longitudinal data cannot thus be carried out directly yet, we modeled a continuous approximation form of the quantile function by using square-root-transformed CD4 count as the response variable. Time since seroconversion, HAART initiation, and baseline characteristics of the patients such as BMI, age, and VL was included in the study. It was found that except age, all the studied variables were found to have a significant effect on CD4 cell counts of HIV-infected patients across all quantiles. Although significant CD4 cell recovery in response to post HAART initiation across all quantiles was recognized, HIV-infected patients who were enrolled in the treatment with a high level of VL showed a significant negative effect on CD4 cell counts at upper quantiles [33]. Even though patients with higher BMI at baseline have improved CD4 cell count overtime after the treatment, they should not be ignored clinically. The study also suggested that physicians should carefully monitor patients with low BMI before and after the treatment because BMI can influence drug metabolism and, consequently, the immunological response to HAART [31, 33]. With the growing recognition of the quantile mixed-effects model, it looks practical that the methodology will be extended to a vast range of statistical applications such as binary data, multi-level models, survival analysis, and other areas of application, and these shall be the subject of future works.

## Supplementary Information

**Additional file 1.** R package *qrLMM()* sample output using CAPRISA 002 Acute Infection Study data across fitted quantile levels.

**Additional file 2.** Graphic overview of convergence for model parameters across all fitted quantiles, produced from the *qrLMM* package using the CAPRISA 002 AI Study data.

## Data Availability

The dataset used for this study can be obtained by requesting Dr. Nonhlanhla Yende-Zuma (Head of Biostatistics Unit, CAPRISA, Email: Nonhlanhla.Yende@caprisa.org) on reasonable request.

## Abbreviations

AI: Acute infection
AIDS: Acquired immune deficiency syndrome
ALD: Asymmetric Laplace distribution
ART: Antiretroviral therapy
ARV: Antiretroviral (drug)
CAPRISA: Centre for the AIDS Programme of Research in South Africa
CD4: Cluster of difference 4 cell (T-lymphocyte cell)
E-step: Expectation step
EM: Expectation–maximization
HAART: Highly Active Antiretroviral Therapy
HIV: Human immunodeficiency virus
LP: Linear programming
M-step: Maximization step
ML: Maximum likelihood
QR: Quantile regression
QR-LMM: Quantile regression for linear mixed-effects models
SAEM: Stochastic Approximation version of the EM algorithm
SE: Standard error
VL: Viral load refers to the number of HIV copies in a milliliter of blood (copies/ml)

## References

[1] AIDSMAP. CD4 cell counts | aidsmap. Key points-May. 2017. https://www.aidsmap.com/about-hiv/cd4-cell-counts.

[2] WHO. Consolidated guidelines on the use of antiretroviral drugs for treating and preventing HIV infection: recommendations for a public health approach. 2016. https://apps.who.int/iris/bitstream/handle/10665/208825/9789241549684_eng.pdf. Accessed 24 Sept 2020. 27466667

[3] Davino C, Furno M, Vistocco D (2013). Quantile regression: theory and applications.

[4] Girma S, Görg H. Foreign direct investment, spillovers and absorptive capacity: evidence from quantile regressions. Bundesbank Series 1 Discussion Paper. 2005.

[5] Chunying Z. A quantile regression analysis on the relations between foreign direct investment and technological innovation in China. In: 2011 international conference of information technology, computer engineering and management sciences, Vol. 4, IEEE. 2011. pp. 38–41.

[6] Mirnezami R, Nicholson J, Darzi A (2012). Preparing for precision medicine. N Engl J Med.

[7] Koenker R, Bassett G (1978). Regression quantiles. Econometrica J Econom Soc.

[8] Pinheiro J, Bates D (2006). Mixed-effects models in S and S-PLUS.

[9] Verbeke G, Molenberghs G (2009). Linear mixed models for longitudinal data.

[10] Twisk JW (2013). Applied longitudinal data analysis for epidemiology: a practical guide.

[11] Diggle P, Diggle PJ, Heagerty P, Liang K-Y, Heagerty PJ, Zeger S (2002). Analysis of longitudinal data.

[12] Brown H, Prescott R (2015). Applied mixed models in medicine.

[13] Koenker R (2005). Quantile regression.

[14] Wichitaksorn N, Choy SB, Gerlach R (2014). A generalized class of skew distributions and associated robust quantile regression models. Can J Stat.

[15] Galvao AF (2011). Quantile regression for dynamic panel data with fixed effects. J Econom.

[16] Fu L, Wang Y-G (2012). Quantile regression for longitudinal data with a working correlation model. Comput Stat Data Anal.

[17] Lipsitz SR, Fitzmaurice GM, Molenberghs G, Zhao LP (1997). Quantile regression methods for longitudinal data with drop-outs: application to CD4 cell counts of patients infected with the human immunodeficiency virus. J R Stat Soc: Ser C (Appl Stat).

[18] Galarza Morales CE. Quantile regression for mixed-effects models. 2015. https://bit.ly/3i7BPyQ.

[19] Geraci M, Bottai M (2007). Quantile regression for longitudinal data using the asymmetric Laplace distribution. Biostatistics.

[20] Geraci M, Bottai M (2014). Linear quantile mixed models. Stat Comput.

[21] Galarza CE, Lachos VH, Bandyopadhyay D (2017). Quantile regression in linear mixed models: a stochastic approximation EM approach. Stat Interface.

[22] Reich BJ (2010). Flexible Bayesian quantile regression for independent and clustered data. Biostatistics.

[23] Noufaily A, Jones M (2013). Parametric quantile regression based on the generalized gamma distribution. J R Stat Soc: Ser C (Appl Stat).

[24] Liu Y, Bottai M (2009). Mixed-effects models for conditional quantiles with longitudinal data. Int J Biostat.

[25] Muir PR, Wallace CC, Done T, Aguirre JD (2015). Limited scope for latitudinal extension of reef corals. Science.

[26] Fornaroli R, Cabrini R, Sartori L, Marazzi F, Vracevic D, Mezzanotte V, Annala M, Canobbio S (2015). Predicting the constraint effect of environmental characteristics on macroinvertebrate density and diversity using quantile regression mixed model. Hydrobiologia.

[27] Blankenberg S, Salomaa V, Makarova N, Ojeda F, Wild P, Lackner KJ, Jørgensen T, Thorand B, Peters A, Nauck M (2016). Troponin I and cardiovascular risk prediction in the general population: the BiomarCaRE Consortium. Eur Heart J.

[28] Patel DE, Geraci M, Cortina-Borja M (2016). Modeling normative kinetic perimetry isopters using mixed-effects quantile regression. J Vis.

[29] Van Loggerenberg F, Mlisana K, Williamson C, Auld SC, Morris L, Gray CM, Karim QA, Grobler A, Barnabas N, Iriogbe I (2008). Establishing a cohort at high risk of HIV infection in South Africa: challenges and experiences of the CAPRISA 002 Acute Infection Study. PLoS ONE.

[30] Mlisana K, Werner L, Garrett NJ, McKinnon LR, van Loggerenberg F, Passmore J-AS, Gray CM, Morris L, Williamson C, Abdool Karim SS (2014). Rapid disease progression in HIV-1 subtype C-infected South African Women. Clin Infect Dis.

[31] Yirga AA, Melesse SF, Mwambi HG, Ayele DG (2020). Modelling CD4 counts before and after HAART for HIV infected patients in KwaZulu-Natal South Africa. Afr Health Sci.

[32] Yirga AA, Melesse SF, Mwambi HG, Ayele DG (2020). Negative binomial mixed models for analyzing longitudinal CD4 count data. Sci Rep.

[33] Yirga AA, Melesse SF, Mwambi HG, Ayele DG (2021). Additive quantile mixed effects modelling with application to longitudinal CD4 count data. Sci Rep.

[34] Whelan D (1999). Gender and HIV/AIDS: taking stock of research and programmes.

[35] UN Women, 2014. Message from UN Women’s Executive Director for World AIDS Day, 1 December 2014. https://www.unwomen.org/en/news/stories/2014/12/world-aids-day-2014.

[36] amfAR. The Foundation for AIDS Research. Statistics: women and HIV/AIDS. 2015. https://www.amfar.org/about-hiv-and-aids/facts-and-stats/statistics--women-and-hiv-aids/.

[37] Kassutto S, Rosenberg ES (2004). Primary HIV type 1 infection. Clin Infect Dis.

[38] Cohen MS, Shaw GM, McMichael AJ, Haynes BF (2011). Acute HIV-1 infection. N Engl J Med.

[39] Rosenberg ES, Altfeld M, Poon SH, Phillips MN, Wilkes BM, Eldridge RL, Robbins GK, Richard T, Goulder PJ, Walker BD (2000). Immune control of HIV-1 after early treatment of acute infection. Nature.

[40] Yirga AA, Ayele DG, Melesse SF (2018). Application of quantile regression: modeling body mass Index in Ethiopia. Open Public Health J.

[41] Buchinsky M (1998). Recent advances in quantile regression models: a practical guideline for empirical research. J Hum Resour.

[42] Ellerbe CN, Gebregziabher M, Korte JE, Mauldin J, Hunt KJ (2013). Quantifying the impact of gestational diabetes mellitus, maternal weight and race on birthweight via quantile regression. PLoS ONE.

[43] Koenker R, Hallock KF (2001). Quantile regression. J Econ Perspect.

[44] Peterson MD, Krishnan C (2015). Growth charts for muscular strength capacity with quantile regression. Am J Prev Med.

[45] Song X, Li G, Zhou Z, Wang X, Ionita-Laza I, Wei Y (2017). QRank: a novel quantile regression tool for eQTL discovery. Bioinformatics.

[46] Sherwood B, Wang L, Zhou XH (2013). Weighted quantile regression for analyzing health care cost data with missing covariates. Stat Med.

[47] Cook BL, Manning WG (2009). Measuring racial/ethnic disparities across the distribution of health care expenditures. Health Serv Res.

[48] Borgoni R (2011). A quantile regression approach to evaluate factors influencing residential indoor radon concentration. Environ Model Assess.

[49] Yu K, Lu Z, Stander J (2003). Quantile regression: Applications and current research areas. J R Stat Soc: Ser D (The Statistician).

[50] Knight CA, Ackerly DD (2002). Variation in nuclear DNA content across environmental gradients: a quantile regression analysis. Ecol Lett.

[51] Cade BS, Noon BR (2003). A gentle introduction to quantile regression for ecologists. Front Ecol Environ.

[52] Lachos VH, Chen M-H, Abanto-Valle CA, Azevedo CL (2015). Quantile regression for censored mixed-effects models with applications to HIV studies. Stat Interface.

[53] Koenker R, Machado JA (1999). Goodness of fit and related inference processes for quantile regression. J Am Stat Assoc.

[54] Cameron AC, Trivedi PK (2005). Microeconometrics: methods and applications.

[55] Cameron AC, Trivedi PK (2009). Microeconometrics using stata.

[56] Cameron AC, Trivedi PK (2013). Regression analysis of count data.

[57] Yu K, Moyeed RA (2001). Bayesian quantile regression. Stat Probab Lett.

[58] Yu K, Zhang J (2005). A three-parameter asymmetric Laplace distribution and its extension. Commun Stat Theory Methods.

[59] Kotz S, Kozubowski T, Podgorski K (2012). The Laplace distribution and generalizations: a revisit with applications to communications, economics, engineering, and finance.

[60] Kozubowski TJ, Nadarajah S (2010). Multitude of Laplace distributions. Stat Pap.

[61] Winkelmann R (2008). Econometric analysis of count data.

[62] Hilbe JM (2011). Negative binomial regression.

[63] Hilbe JM (2014). Modeling count data.

[64] Machado JAF, Silva JS (2005). Quantiles for counts. J Am Stat Assoc.

[65] Winkelmann R (2006). Reforming health care: evidence from quantile regressions for counts. J Health Econ.

[66] Miranda A (2008). Planned fertility and family background: a quantile regression for counts analysis. J Popul Econ.

[67] Dempster AP (1977). Maximum likelihood from incomplete data via the EM algorithm. J R Stat Soc: Ser B (Methodol).

[68] McLachlan GJ, Krishnan T (2007). The EM algorithm and extensions.

[69] Delyon B, Lavielle M, Moulines E (1999). Convergence of a stochastic approximation version of the EM algorithm. Ann Stat.

[70] Jank W (2006). Implementing and diagnosing the stochastic approximation EM algorithm. J Comput Graph Stat.

[71] Meza C, Osorio F, De la Cruz R (2012). Estimation in nonlinear mixed-effects models using heavy-tailed distributions. Stat Comput.

[72] Kuhn E, Lavielle M (2004). Coupling a stochastic approximation version of EM with an MCMC procedure. ESAIM Probab Stat.

[73] Kuhn E, Lavielle M (2005). Maximum likelihood estimation in nonlinear mixed effects models. Comput Stat Data Anal.

