# Supplementary material for: Application of quantile mixed-effects model in modeling CD4 count from HIV-infected patients in KwaZulu-Natal South Africa
Source: BMC Infect Dis. 2022 Jan 4;22:20. doi: 10.1186/s12879-021-06942-7 (PMC8724661; doi:10.1186/s12879-021-06942-7)
Supplement: Supplementary file 1 — Additional file 1. R package qrLMM() sample output using CAPRISA 002 Acute Infection Study data across fitted quantile levels. [file 12879_2021_6942_MOESM1_ESM.pdf]

Application of quantile mixed-effects model in modeling CD4 count from HIV-infected patients in KwaZulu-Natal, South Africa

Ashenafi A Yirga[1*], Sileshi F Melesse[1], Henry G Mwambi[1] and Dawit G Ayele[2]

1. School of Mathematics, Statistics, and Computer Science, University of KwaZulu-Natal, Pietermaritzburg, Private Bag X01, Scottsville, 3209, South Africa.
2. Institute of Human Virology, University of Maryland, School of Medicine, USA.

*Corresponding Author: Ashenafi Argaw Yirga, Ph.D. Candidate
University of KwaZulu-Natal
Pietermaritzburg, South Africa
Email: ashu3argaw@gmail.com, or 216065934@stu.ukzn.ac.za

Additional file1: R package *qrLMM()* sample outputs using CAPRISA 002 Acute Infection Study data across all fitted quantile levels.

```
---------------------------------------------------
Quantile Regression for Linear Mixed Model
---------------------------------------------------

Quantile = 0.05 0.25 0.5 0.75 0.85 0.95
Subjects = 235 ; Observations = 7019

-----------
Estimates
-----------

- Fixed effects

       Estimate Std. Error Inf CI95% Sup CI95%  z value Pr(>|z|)
beta 1 19.99675    1.16072  17.72173  22.27177 17.22783  0.00000
beta 2  0.06294    0.01473   0.03407   0.09180  4.27392  0.00002
beta 3 -0.86567    0.14159  -1.14319  -0.58815 -6.11395  0.00000
beta 4  0.05642    0.02067   0.01590   0.09694  2.72903  0.00635
beta 5 -0.56383    0.07799  -0.71670  -0.41096 -7.22912  0.00000
beta 6  1.68287    0.05379   1.57744   1.78830 31.28520  0.00000
beta 7  0.02073    0.02500  -0.02826   0.06972  0.82939  0.40688


ii) Variance-Covariance Matrix
         z1       z2       z3
z1 24.80823  0.22416 -3.39361
z2  0.22416  0.03539 -0.26853
z3 -3.39361 -0.26853  2.72345

------------------------
Model selection criteria
------------------------

         Loglik      AIC      BIC       HQ
Value -18454.68 36937.36 37033.35 36970.43

-------
Details
-------

Convergence reached? = TRUE
Iterations = 418 / 500
Criteria = 0.00046
MC sample = 20
Cut point = 0.25
Processing time = 2.725659 hours
```

Quantile Regression for Linear Mixed Model

Quantile = 0.05 **0.25** 0.5 0.75 0.85 0.95

Subjects = 235 ; Observations = 7019

Estimates

- Fixed effects

	Estimate	Std. Error	Inf CI95%	Sup CI95%	z value	Pr(>|z|)
beta 1	22.17136	1.40335	19.42079	24.92193	15.79884	0.00000
beta 2	0.06979	0.01369	0.04296	0.09661	5.09845	0.00000
beta 3	-0.87112	0.12946	-1.12486	-0.61738	-6.72888	0.00000
beta 4	0.07836	0.02432	0.03070	0.12602	3.22263	0.00127
beta 5	-0.56874	0.10345	-0.77149	-0.36598	-5.49793	0.00000
beta 6	2.12541	0.07329	1.98176	2.26907	28.99856	0.00000
beta 7	0.02957	0.02972	-0.02868	0.08781	0.99496	0.31975

sigma = 0.68658

Random effects Variance-Covariance Matrix matrix

	z1	z2	z3
z1	17.94444	0.15706	-2.85704
z2	0.15706	0.02222	-0.19180
z3	-2.85704	-0.19180	2.12359

Model selection criteria

	Loglik	AIC	BIC	HQ
Value	-17169.85	34367.69	34463.68	34400.77

Details

Convergence reached? = TRUE

Iterations = 477 / 500

Criteria = 0.00041

MC sample = 20

Cut point = 0.25

Processing time = 2.915623 hours

```
---------------------------------------------------
"Quantile Regression for Linear Mixed Model
---------------------------------------------------
Quantile = 0.05 0.25 0.5 0.75 0.85 0.95
Subjects = 235; Observations = 7019
-----------
Estimates
-----------
- Fixed effects

       Estimate Std. Error Inf CI95% Sup CI95%  z value Pr(>|z|)
beta 1 24.62849    1.46389  21.75927  27.49770 16.82404  0.00000
beta 2  0.05678    0.01285   0.03159   0.08197  4.41807  0.00001
beta 3 -0.69589    0.11792  -0.92702  -0.46477 -5.90142  0.00000
beta 4  0.08170    0.02699   0.02879   0.13461  3.02638  0.00248
beta 5 -0.64095    0.09631  -0.82972  -0.45219 -6.65513  0.00000
beta 6  2.56004    0.08808   2.38740   2.73268 29.06490  0.00000
beta 7  0.02935    0.03125  -0.03191   0.09060  0.93898  0.34774

sigma = 0.87713

Random effects Variance-Covariance Matrix
         z1       z2       z3
z1 17.69164  0.16071 -2.93488
z2  0.16071  0.02010 -0.18231
z3 -2.93488 -0.18231  2.03916

------------------------
Model selection criteria
------------------------
         Loglik      AIC      BIC       HQ
Value -16828.96 33685.92 33781.91 33718.99
-------
Details
-------
Convergence reached? = TRUE
Iterations = 433 / 500
Criteria = 0.00036
MC sample = 20
Cut point = 0.25
Processing time = 2.591965 hours"
```

```
---------------------------------------------------
Quantile Regression for Linear Mixed Model
---------------------------------------------------

Quantile = 0.05 0.25 0.5 0.75 0.85 0.95
Subjects = 235 ; Observations = 7019

-----------
Estimates
-----------

- Fixed effects

       Estimate Std. Error Inf CI95% Sup CI95%  z value Pr(>|z|)
beta 1 26.59571    1.41986  23.81279  29.37863 18.73127  0.00000
beta 2  0.04600    0.01321   0.02011   0.07189  3.48205  0.00050
beta 3 -0.59311    0.11969  -0.82770  -0.35852 -4.95549  0.00000
beta 4  0.11195    0.03207   0.04910   0.17480  3.49109  0.00048
beta 5 -0.71365    0.09390  -0.89770  -0.52960 -7.59987  0.00000
beta 6  3.02180    0.09680   2.83208   3.21152 31.21830  0.00000
beta 7  0.02988    0.03154  -0.03194   0.09170  0.94725  0.34351

sigma = 0.70722

Random effects Variance-Covariance Matrix matrix
         z1       z2       z3
z1 19.23673  0.11625 -2.57316
z2  0.11625  0.01980 -0.18194
z3 -2.57316 -0.18194  2.04732

------------------------
Model selection criteria
------------------------

         Loglik      AIC      BIC       HQ
Value -17344.63 34717.25 34813.24 34750.33

-------
Details
-------

Convergence reached? = FALSE
Iterations = 500 / 500
Criteria = 5e-04
MC sample = 20
Cut point = 0.25
Processing time = 4.430015 hours
Press [ENTER] to see next plot:
```

```
---------------------------------------------------
Quantile Regression for Linear Mixed Model
---------------------------------------------------

Quantile = 0.05 0.25 0.5 0.75 0.85 0.95
Subjects = 235 ; Observations = 7019

-----------
Estimates
-----------

- Fixed effects

       Estimate Std. Error Inf CI95% Sup CI95%  z value Pr(>|z|)
beta 1 27.97228    1.42043  25.18824  30.75631 19.69286  0.00000
beta 2  0.04132    0.01313   0.01559   0.06705  3.14764  0.00165
beta 3 -0.58146    0.12470  -0.82587  -0.33705 -4.66292  0.00000
beta 4  0.13131    0.03383   0.06500   0.19762  3.88101  0.00010
beta 5 -0.71471    0.08997  -0.89105  -0.53837 -7.94381  0.00000
beta 6  3.11409    0.09728   2.92342   3.30476 32.01100  0.00000
beta 7  0.02576    0.03215  -0.03725   0.08878  0.80134  0.42294

sigma = 0.52207
ii) Variance-Covariance Matrix
         z1       z2       z3
z1 23.36092  0.04613 -2.18991
z2  0.04613  0.02165 -0.19618
z3 -2.18991 -0.19618  2.17872

------------------------
Model selection criteria
------------------------

        Loglik   AIC      BIC       HQ
Value -17952.5 35933 36028.99 35966.07

-------
Details
-------

Convergence reached? = FALSE
Iterations = 500 / 500
Criteria = 0.00065
MC sample = 20
Cut point = 0.25
Processing time = 3.066678 hours
```

```
---------------------------------------------------
Quantile Regression for Linear Mixed Model
---------------------------------------------------

Quantile = 0.05 0.25 0.5 0.75 0.85 0.95
Subjects = 235; Observations = 7019

-----------
Estimates
-----------

- Fixed effects

       Estimate Std. Error Inf CI95% Sup CI95%  z value Pr(>|z|)
beta 1 31.38118    1.39665  28.64373  34.11862 22.46882  0.00000
beta 2  0.03366    0.01578   0.00273   0.06459  2.13293  0.03293
beta 3 -0.38521    0.16218  -0.70309  -0.06734 -2.37520  0.01754
beta 4  0.14515    0.03043   0.08551   0.20480  4.76972  0.00000
beta 5 -0.73982    0.08494  -0.90631  -0.57333 -8.70959  0.00000
beta 6  2.28722    0.08890   2.11298   2.46146 25.72899  0.00000
beta 7  0.01328    0.03032  -0.04615   0.07271  0.43812  0.66130

sigma = 0.2237


 ii) Variance-Covariance Matrix
          z1       z2       z3
 z1 33.52005  0.02446 -2.84757
 z2  0.02446  0.03175 -0.29260
 z3 -2.84757 -0.29260  3.29493

 ------------------------
 Model selection criteria
 ------------------------

          Loglik      AIC      BIC       HQ
 Value -19088.77 38205.55 38301.54 38238.62

 -------
 Details
 -------

 Convergence reached? = FALSE
 Iterations = 500 / 500
 Criteria = 0.00153
 MC sample = 20
 Cut point = 0.25
 Processing time = 3.169162 hours
```
