# Supplementary material for: Application of quantile mixed-effects model in modeling CD4 count from HIV-infected patients in KwaZulu-Natal South Africa
Source: BMC Infect Dis. 2022 Jan 4;22:20. doi: 10.1186/s12879-021-06942-7 (PMC8724661; doi:10.1186/s12879-021-06942-7)

Additional file2: Graphic overview of convergence for model parameters across all fitted quantiles, produced from the *qrLMM* package using the CAPRISA 002 AI Study data.

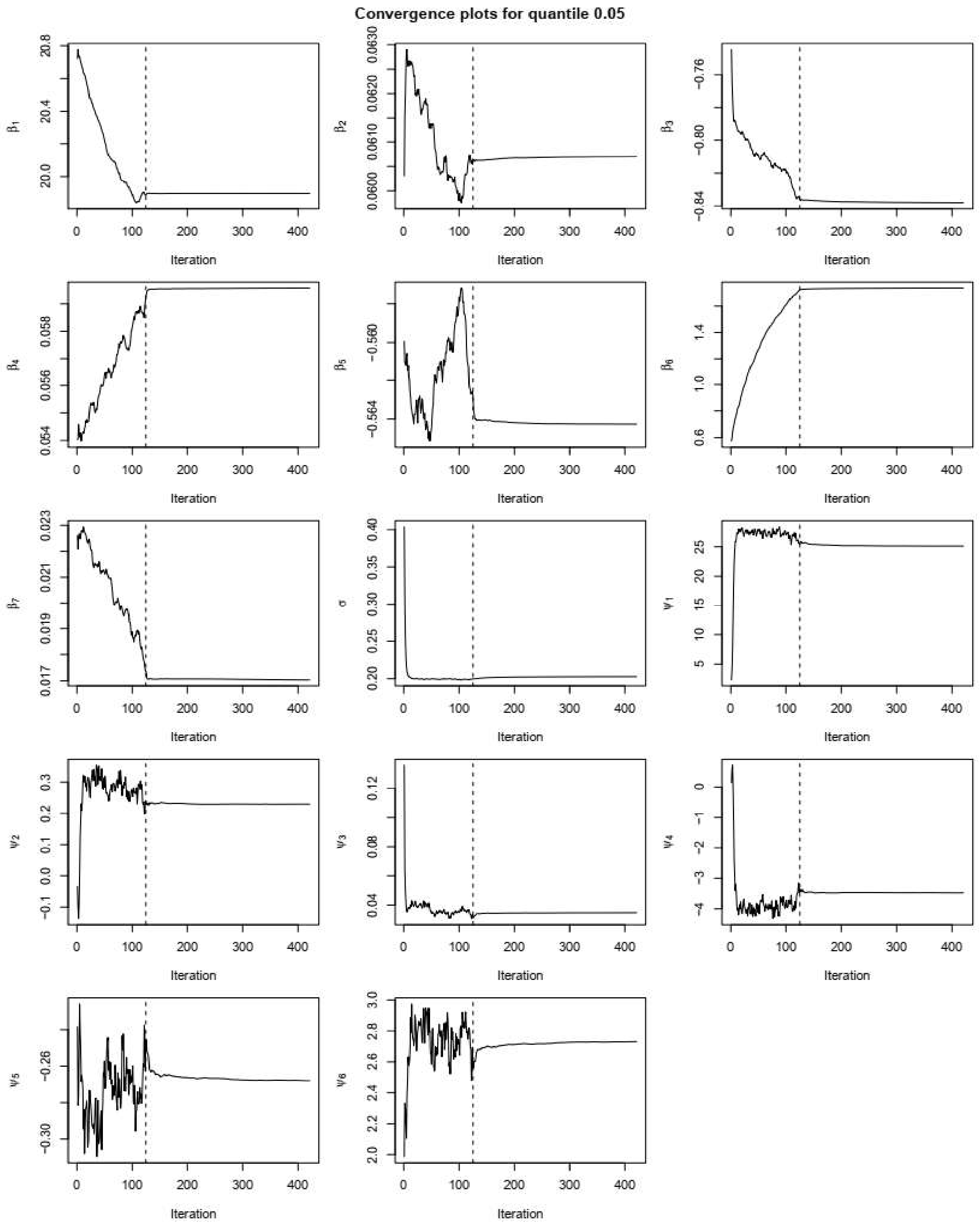

Convergence plots for quantile 0.25

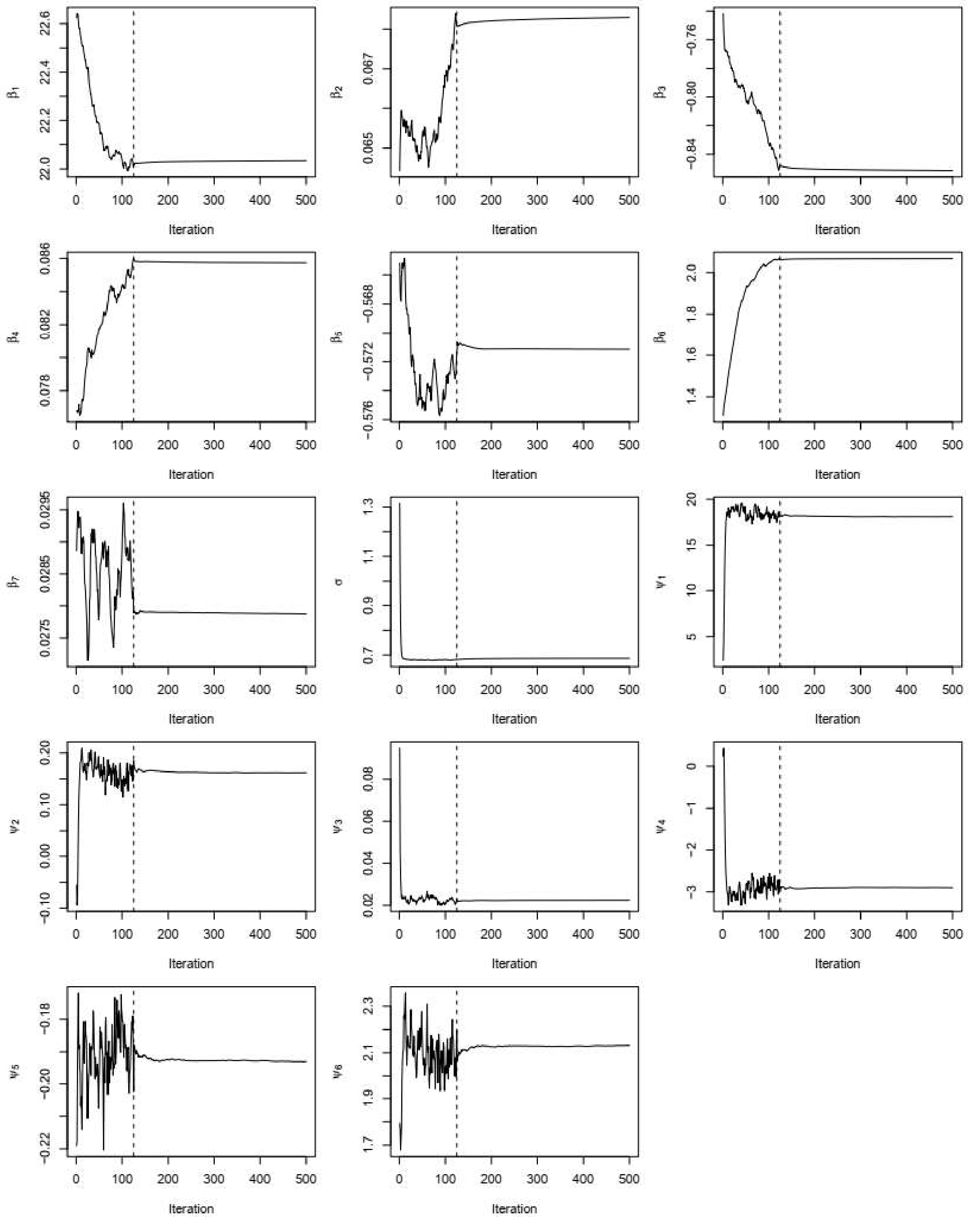

Convergence plots for quantile 0.5

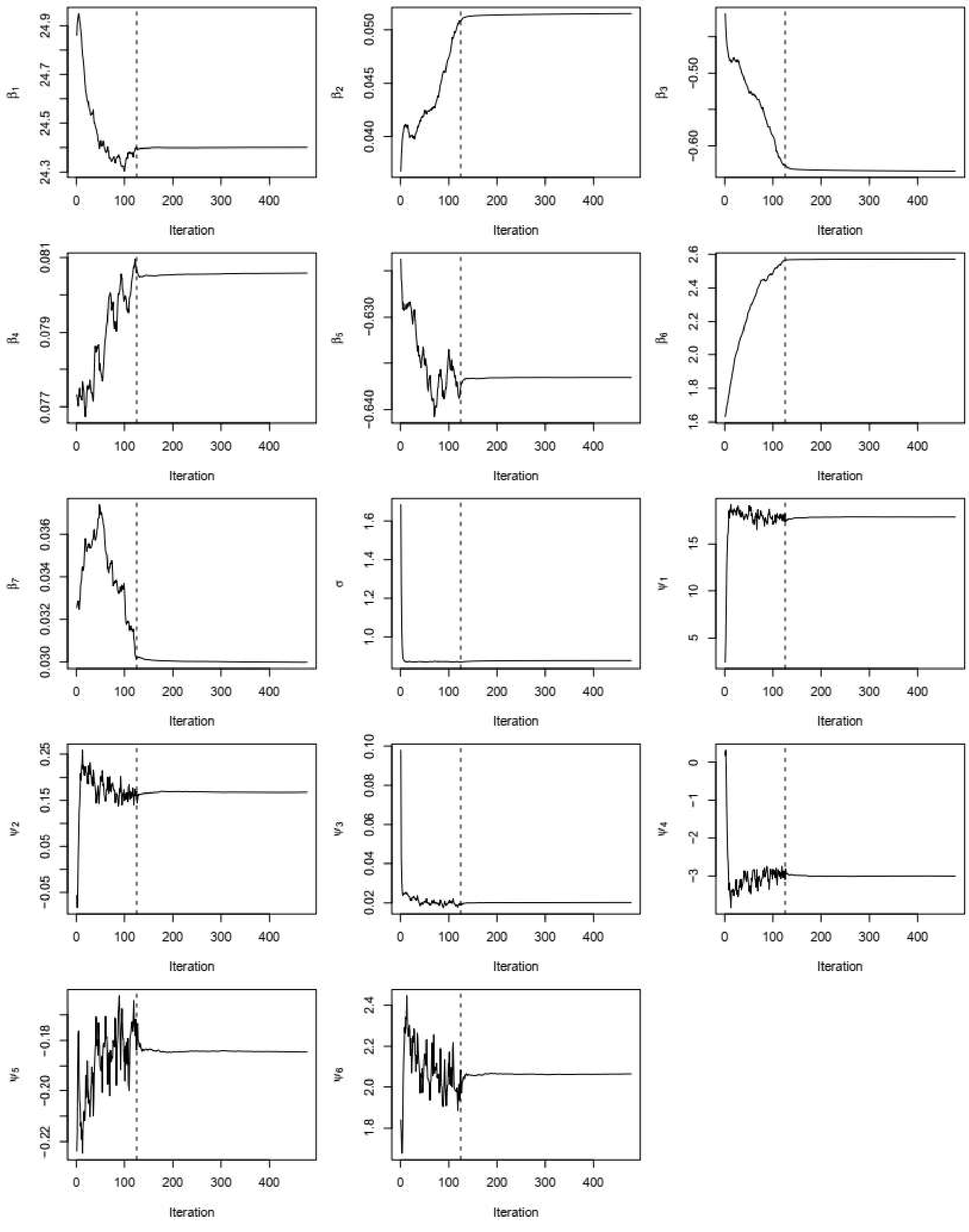

Convergence plots for quantile 0.75

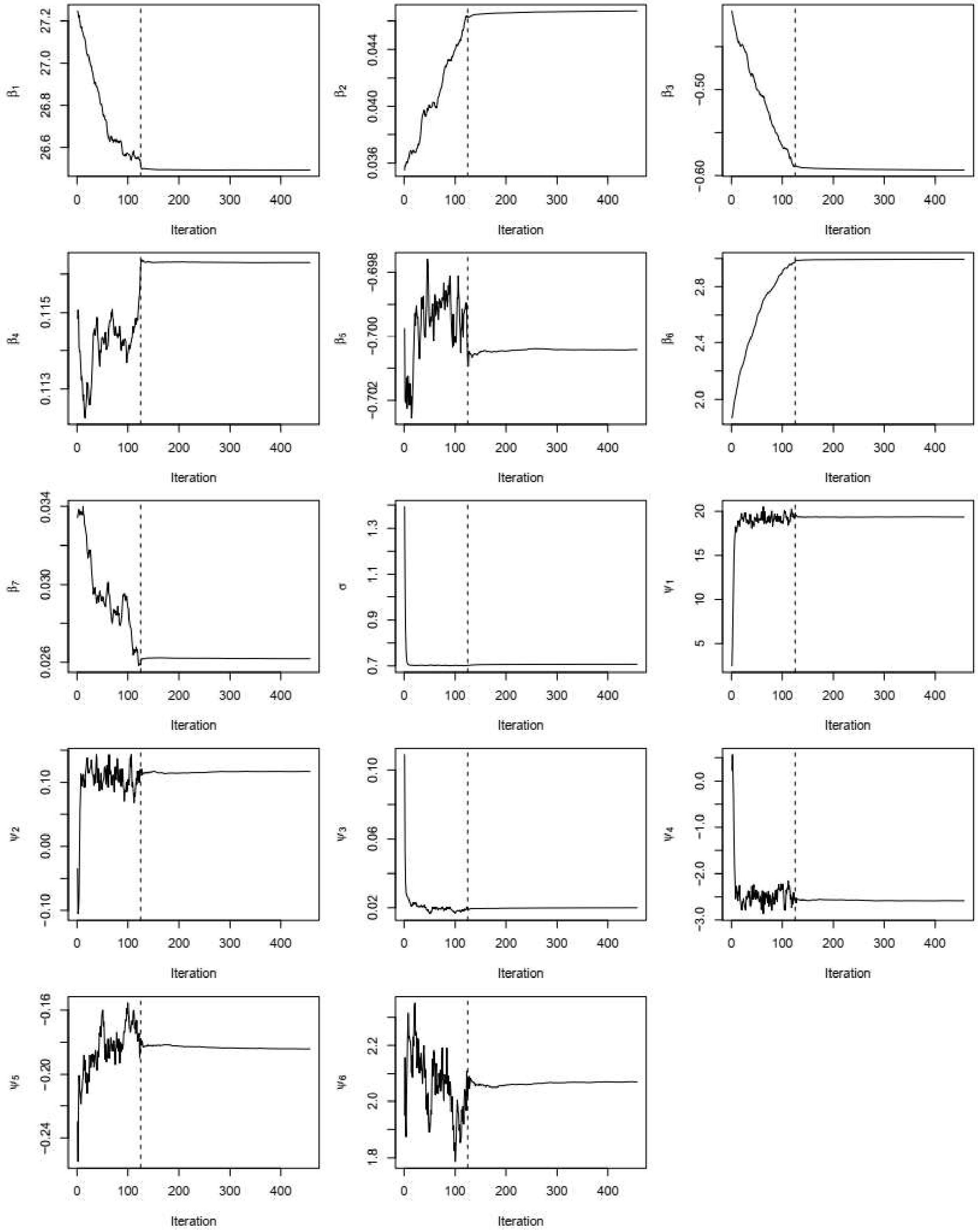

Convergence plots for quantile 0.85

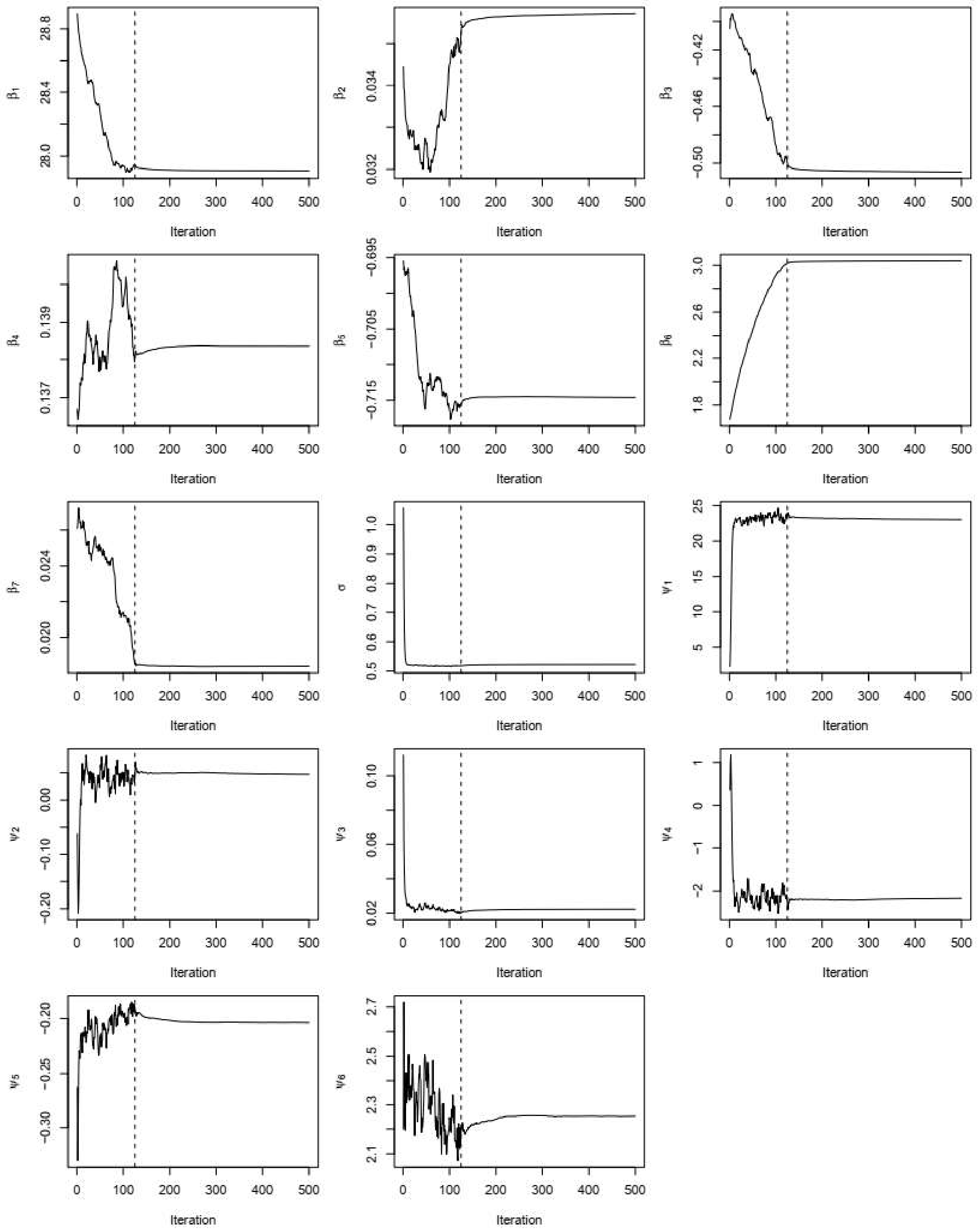

Convergence plots for quantile 0.95

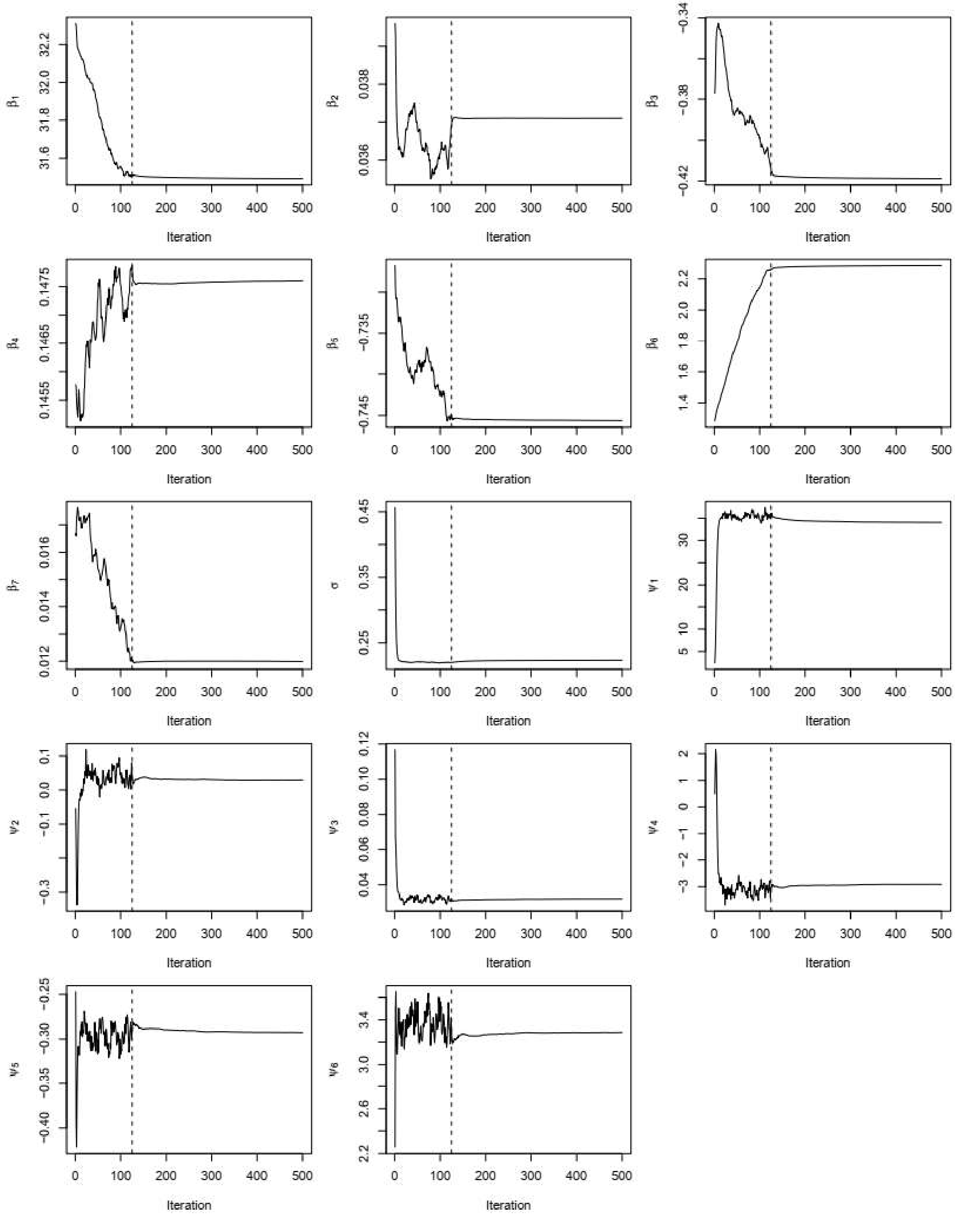

Supplement: Supplementary file 2 — Additional file 2. Graphic overview of convergence for model parameters across all fitted quantiles, produced from the qrLMM package using the CAPRISA 002 AI Study data. [file 12879_2021_6942_MOESM2_ESM.pdf]
